# Cost-effectiveness analysis of a first-line treatment with cadonilimab plus platinum-based chemotherapy with or without bevacizumab for persistent, recurrent, or metastatic cervical cancer in China: COMPASSION-16 trial

**DOI:** 10.1080/20523211.2025.2464781

**Published:** 2025-02-17

**Authors:** Yiling Ding, Chunping Wang, Yamin Shu, Jinglin Wang, Qilin Zhang

**Affiliations:** a Department of Pharmacy, Tongji Hospital, Tongji Medical College, Huazhong University of Science and Technology, Wuhan, People’s Republic of China; b International Research Center for Medicinal Administration, Peking University, Beijing, People’s Republic of China; c School of Pharmaceutical Sciences, Peking University, Beijing, People’s Republic of China; d Department of Pharmacy, Union Hospital, Tongji Medical College, Huazhong University of Science and Technology, Wuhan, People’s Republic of China

**Keywords:** Cadonilimab, cervical cancer, cost-effectiveness, partitioned survival model, COMPASSION-16 trial

## Abstract

**Background:**

The addition of cadonilimab to first-line platinum-based chemotherapy with or without bevacizumab significantly improved progression-free survival (PFS) and overall survival (OS) in patients with persistent, recurrent, or metastatic cervical cancer. However, the economic value of using this novel therapy for this indication is currently unknown. The aim of this study is to evaluate the cost-effectiveness of the addition of cadonilimab to first-line standard chemotherapy for patients with persistent, recurrent, or metastatic cervical cancer from the perspective of Chinese healthcare system.

**Methods:**

A partitioned survival model was constructed to compare the cost-effectiveness of cadonilimab versus placebo in patients enrolled in the COMPASSION-16 trial. Cost, life-year, quality-adjusted life-year (QALY), incremental cost-effectiveness ratio (ICER), incremental net health benefit (INHB), and incremental net monetary benefit (INMB) were calculated for 2 treatment strategies. Sensitivity, scenario, and subgroup analyses, and value of information analysis (EVPI) were performed.

**Results:**

Cadonilimab provided an additional 1.18 QALYs and $89,528.64 compared with placebo, which resulted in an ICER of $75,944.56/QALY. At the willingness-to-pay threshold of $38,042.49/QALY, INHB was estimated to be −1.17 QALYs, while INMB amounted to −$44,681.55 and EVPI was calculated as $71.40/person. Sensitivity analyses revealed that the model was most sensitive to hazard ratio (HR) for OS and PFS, and the probability of cadonilimab being cost-effective was 0.70%. To achieve cost-effectiveness, the price of cadonilimab must be reduced by approximately 50%. Subgroup analysis found that all subgroups unfavored cadonilimab by varying the HR for OS and PFS. Scenario analyses showed using life-year as effectiveness, altering time horizon and selection of survival analysis did not reverse results.

**Conclusions:**

Although the use of cadonilimab resulted in clinical benefit, it was not deemed cost-effective as a first-line therapy for persistent, recurrent, or metastatic cervical cancer in China. Lowering the price of cadonilimab may enhance its cost-effectiveness.

## 1. Introduction

Cervical cancer is the fourth leading cause of cancer death among women worldwide, accounting for more than 660,000 diagnosed cases and 340,000 deaths each year (Bray et al., 2024). According to the latest data, 150,700 new cervical cancer cases occurred in China, resulting in an estimated 55,700 deaths in 2022 (Han et al., 2024). The high mortality rates associated with cervical cancer contribute to a significant global disease burden. The prognosis remains poor for those with recurrent or metastatic disease, evidenced by a 5-year relative survival rate of 19% (Cervical Cancer Prognosis and Survival Rates, 2023).

For over a decade, the standard first-line treatment for patients with recurrent or metastatic cervical cancer was platinum-based chemotherapy (Abu-Rustum et al., 2023). The GOG 240 study showed that adding bevacizumab to systemic chemotherapy modestly improved OS to 16.8 months (Tewari et al., 2017). Hence, the National Comprehensive Cancer Network (NCCN) recommends the combination of chemotherapy and bevacizumab as the preferred first-line therapy for patients with recurrent or metastatic cervical cancer (Abu-Rustum et al., 2023). Recently, accumulating high-quality evidence strengthens the role of various immunotherapies, including PD-1/PD-L1 inhibitors, CTLA-4 inhibitors, combined with chemotherapy, with or without bevacizumab, in improving outcomes in patients with metastatic or recurrent cervical cancer (Fang et al., 2024; Monk et al., 2023; O'Malley et al., 2022; Oaknin et al., 2024). Based on the promising results of KEYNOTE-826 trial, pembrolizumab in combination with chemotherapy has been approved by FDA in 2021 as the preferred first-line therapy for patients who are PD-L1 positive, because of a significant improvement in OS to approximately 26 months (Monk et al., 2023). In addition, the BEATcc study assessed that the PD-L1 antibody atezolizumab combined with bevacizumab into first-line chemotherapy resulted in a substantial extension of median OS of 32.1 months (Oaknin et al., 2024). However, as a limitation, the trials either excluded participants with contraindications to bevacizumab or included mostly white participants with few or no Asian individuals. Novel treatments are needed to improve safety in these population and preserve the efficacy of the combination.

Cadonilimab is a novel bispecific antibody blocking PD-1 and CTLA-4 pathways simultaneously (Pang et al., 2023). Recently, the COMPASSION-16 multicentre phase 3 randomized clinical trial first revealed that the addition of cadonilimab to first-line platinum-based chemotherapy with or without bevacizumab significantly prolonged the median PFS (12.7 months vs. 8.1 months, HR 0.62; 95% CI, 0.49–0.80; *P* < 0.0001) compared with placebo plus chemotherapy for individuals who are PD-L1 positive and negative (Wu et al., 2024). The median OS was not reached versus 22.8 months, respectively (HR 0.64; 95% CI, 0.48–0.86; *P* = 0.0011). The rate of grade 3 or higher treatment-related adverse events (AEs) was comparable between the 2 groups (82% vs. 79%). Compared with KEYNOTE-826 and BEATcc studies, the COMPASSION-16 trial enrolled participants regardless of PD-L1 expression status, and treatment options included combination or no combination of bevacizumab, expanding the population of participants and adding more evidence in Asian population. As a bispecific antibody, cadonilimab is revolutionizing the standard treatment approach for cervical cancer, particularly when compared to single-target antibodies. Thus, cadonilimab plus chemotherapy regimen seemed to be an attractive alternative for the first-line treatment of persistent, recurrent, or metastatic cervical cancer (Romero, 2025).

Cadonilimab has undoubtedly provided crucial evidence for updating cervical cancer treatment guidelines. However, it is necessary to acknowledge that the addition of cadonilimab significantly increases the cost of treatment and imposes a substantial economic burden on patients and society, especially in a developing country like China. At present, there is no evidence of the economic value of this novel therapy. Considering cost-effectiveness is helpful for clinicians and decision-makers to evaluate the accessibility of this immunotherapy combination to the public. This study aimed to investigate the cost-effectiveness of cadonilimab plus standard chemotherapy with or without bevacizumab as first-line treatment for persistent, recurrent, or metastatic cervical cancer from the perspective of the Chinese healthcare system.

## 2. Methods

### 2.1. Model construction

**Figure 1.**
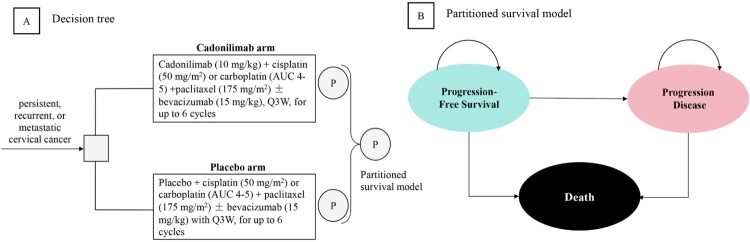
Model structure of a decision tree combining the partitioned survival model with 3 health state.

We performed a partitioned survival model (PSM) with 3 mutually exclusive health states (Figure 1), the most common cost-effectiveness analysis (CEA) model used for advanced oncologic therapies (Woods et al., 2020). The PFS and OS curves were utilized for the computation of the proportions pertaining to states, which collectively sum up to 1. The proportion of PD was estimated by calculating the difference in area under curves between the OS and PFS, and the fraction of deceased patients could be calculated as 1 minus the survival rate (OS) (Pahuta et al., 2019; Woods et al., 2020). The time horizon for the study was set at 30 years, with a cycle length of 1 week. The study population for this analysis included women aged 18–75 years with persistent, recurrent, or metastatic (stage IVB) cervical cancer of squamous cell carcinoma, adenocarcinoma, or adenosquamous carcinoma. These individuals had not received any previous systemic therapy and their characteristics were consistent with those of patients enrolled in the COMPASSION-16 trial (Wu et al., 2024). Additional criteria for inclusion and exclusion were provided in detail within the trial. A total of approximately 440 subjects were enrolled in this trial and randomly assigned to two groups in a 1:1 ratio. This economic analysis was based on a literature review and modeling techniques and did not require approval from an Institutional Research Ethics Board.

During the follow-up period, the GetData Graph Digitizer was used to capture graphic data from the PFS and OS curves in the COMPASSION-16 trial. The unknown survival benefit was estimated by fitting and extrapolating the data points using various parametric survival functions, including Exponential, Gamma, Generalized gamma, Gompertz, Weibull, Log-logistic, Log-normal, and Royston-Parmar (L. Liu et al., 2023). We selected survival functions that fitted best based on the lowest value of the Akaike information criterion (AIC) and Bayesian information criterion (BIC), visual inspection, and the expert opinion of the oncologist (Latimer, 2013). The results of the goodness-of-fit analysis were presented in Supplemental Table S1. The PFS distribution and OS distribution were Royston-Parmar and Log-logistic for cadonilimab group, and Royston-Parmar and Log-normal for placebo group (Table 1). The validation plot and survival distribution were shown in Supplemental Figures S1 and S2. To mitigate the bias arising from inconsistencies in parametric survival distribution between the two groups, we employed the proportional hazards (PH) modeling approach to generate the parametric PFS and OS curves for the cadonilimab group based on hazard ratios (HRs) derived from the COMPASSION-16 trial comparing cadonilimab with placebo (Latimer, 2013; Wu et al., 2024). The fitting trend of PH and parametric survival functions for cadonilimab were shown in Supplemental Figure S3. This CEA study followed the Consolidated Health Economic Evaluation Reporting Standards (CHEERS) reporting guideline (Husereau et al., 2022). The survival curves were fitted using the flexsurv package in R software, while the PSM was constructed utilizing Microsoft Excel 2021.

**Table 1.** Key model inputs.

Parameter	Base case (Range)	Distribution	Source
**Clinical survival**			
Royston-Parmar for Cadonilimab PFS	gamma0 = −5.2107, gamma1 = 1.9436, gamma2 = −0.6438, gamma3 = 1.0688		
Royston-Parmar for Placebo PFS	gamma0 = −4.9688, gamma1 = 2.1035, gamma2 = −1.0286, gamma3 = 1.3374		
Log-logistic for Cadonilimab OS	shape = 1.6517, scale = 32.3136		
Log-normal for Placebo OS	meanlog = 3.1181, sdlog = 0.9163		
HR for OS	0.64 (0.48–0.86)	Log-normal	(Wu et al., 2024)
HR for PFS	0.62 (0.49–0.79)	Log-normal	(Wu et al., 2024)
**Costs ($)**			
Cost of Cadonilimab per 125mg	875.02 (700.02–875.02)	Gamma	(Chinese Drug Price of Drug Centralized Bid Procurement, 2024)
Cost of Carboplatin per 100mg	7.32 (5.86–7.32)	Gamma	(Chinese Drug Price of Drug Centralized Bid Procurement, 2024)
Cost of Cisplatin per 20mg	2.43 (1.95–2.43)	Gamma	(Chinese Drug Price of Drug Centralized Bid Procurement, 2024)
Cost of Paclitaxel per 100mg	26.32 (21.06–26.32)	Gamma	(Chinese Drug Price of Drug Centralized Bid Procurement, 2024)
Cost of Bevacizumab per 100mg	158.37 (126.7–158.37)	Gamma	(Chinese Drug Price of Drug Centralized Bid Procurement, 2024)
Cost of intravenous drug administration	44.43 (35.54–53.32)	Gamma	(Lin et al., 2024)
Cost of tumor imaging per unit	99.34 (79.47–119.2)	Gamma	Local medical
Cost of laboratory testing per unit	56.76 (45.41–68.12)	Gamma	Local medical
Cost of terminal care in end-of-life	1460.3 (1168.24–1752.36)	Gamma	(Liu et al., 2023)
Cost of Anaemia	531.7 (425.36–638.04)	Gamma	(Liu et al., 2023)
Cost of White blood cell count decreased	466 (372.8–559.2)	Gamma	(Liu et al., 2023)
Cost of Neutrophil count decreased	461.5 (369.2–553.8)	Gamma	(Liu et al., 2023)
Cost of Platelet count decreased	1,054.22 (843.38–1,265.06)	Gamma	(Wen et al., 2021)
Cost of Urinary tract infection	126.03 (100.82–151.24)	Gamma	(Lin et al., 2024)
Cost of Hypokalaemia	15.8 (12.64–18.96)	Gamma	(Liu et al., 2023)
**Incidence of 3–5 grade adverse events**			(Wu et al., 2024)
Propability of Anaemia in Cadonilimab	0.17 (0.14–0.2)	Beta	
Propability of Anaemia in Placebo	0.26 (0.21–0.31)	Beta	
Propability of White blood cell count decreased in Cadonilimab	0.28 (0.22–0.34)	Beta	
Propability of White blood cell count decreased in Placebo	0.36 (0.29–0.43)	Beta	
Propability of Neutrophil count decreased in Cadonilimab	0.41 (0.33–0.49)	Beta	
Propability of Neutrophil count decreased in Placebo	0.46 (0.37–0.55)	Beta	
Propability of Platelet count decreased in Cadonilimab	0.14 (0.11–0.17)	Beta	
Propability of Platelet count decreased in Placebo	0.12 (0.1–0.14)	Beta	
Propability of Urinary tract infection in Cadonilimab	0.06 (0.05–0.07)	Beta	
Propability of Urinary tract infection in Placebo	0.05 (0.04–0.06)	Beta	
Propability of Hypokalaemia in Cadonilimab	0.06 (0.05–0.07)	Beta	
Propability of Hypokalaemia in Placebo	0.05 (0.04–0.06)	Beta	
**Utility**			
Utility of PFS	0.76 (0.61–0.91)	Beta	(Lin et al., 2024)
Utility of PD	0.52 (0.42–0.62)	Beta	(Lin et al., 2024)
Disutility of Grade 3–5 AEs	0.2 (0.16–0.24)	Beta	(Liu et al., 2023)
**Others**			
Body surface area, m^2^	1.72 (1.38–2.06)	Log-normal	(Liu et al., 2023)
Weight, kg	59.8 (47.84–71.76)	Log-normal	(Report of Healthy Height and Weight of Chinese Residents in 2022, 2022)
Discount rate	0.05 (0.04–0.06)	Beta	(Liu et al., 2020)

Abbreviations: HR, hazard ratio; AEs, adverse events; PFS, progression-free survival; PD, progressed disease.

### 2.2. Cost and utility inputs

The calculation of costs in this CEA focused exclusively on direct medical expenses incurred within the healthcare system (Table 1). These encompassed medication costs, expenses related to tumor imaging and laboratory testing, terminal care and adverse event management, as well as other medical expenditures. The AEs ≥ grade 3 with an incidence rate exceeding 5% were incorporated into the model, and assumed to occur during the initial cycle. The costs were converted to 2023 US dollars using an exchange rate of $1 = ¥7.05 (China Foreign Exchange Trade System, 2024). The costs of the medicine were derived from the winning bid price in China (Chinese Drug Price of Drug Centralized Bid Procurement, 2024), while other cost data were obtained from published literature (Lin et al., 2024; L. Liu et al., 2023; Wen et al., 2021).

The costs of first-line therapeutic drugs were determined based on the dosage, duration, and longest sustained period utilized in COMPASSION-16 trial. We assumed that a typical Chinese female had an average body surface area of 1.72 m^2^ with 59.8 Kg (L. Liu et al., 2023; Report of Healthy Height and Weight of Chinese Residents in 2022, 2022). As delineated in the trial (Wu et al., 2024), eligible patients received cadonilimab (10 mg/kg) or placebo in combination with chemotherapy, comprising cisplatin (50 mg/m², 92/226 in cadonilimab and 100/219 in placebo) or carboplatin (area under the curve/AUC 4-5, 134/226 in cadonilimab and 119/219 in placebo), along with paclitaxel (175 mg/m²), either with or without bevacizumab (15 mg/kg, 135/226 in cadonilimab and 129/219 in placebo). The treatment protocol was administered in 3-week intervals for a maximum of six cycles, followed by maintenance therapy with cadonilimab or placebo, with or without bevacizumab, also given every three weeks. The treatment was continued until disease progression, unacceptable toxicity, or completion of a 2-year period of receiving cadonilimab or placebo. Considering the absence of specific treatment plans following disease progression in the trial, we presumed that patients underwent paclitaxel therapy.

The COMPASSION-16 trial did not provide utility values. However, a cost-effectiveness analysis involving cervical cancer patients reported PFS and PD rates of 0.76 and 0.52, respectively, for cervical cancer (Lin et al., 2024). Moreover, the disutility values associated with AEs ≥ grade 3 (>5%) were incorporated into this CEA (L. Liu et al., 2023), and the baseline PFS utility was adjusted by subtracting the disutility adjusted for duration.

### 2.3. Base-case analysis

We chose the PH model in the PSM as our base-case analysis and initially conducted a CEA. The clinical and economic outcomes were compared between the cadonilimab group and placebo group, encompassing measures such as average life-years, quality-adjusted life-year (QALY), and costs. The incremental cost-effectiveness ratio (ICER) was calculated as the additional costs per gained QALY between the two groups. If the ICER fell below the pre-specified willingness-to-pay threshold (WTP), which is three times China's GDP per capita per QALY ($38,042.49/QALY), the cadonilimab strategy was deemed cost-effective in accordance with the recommendations outlined in the Guidelines for Evaluation of Chinese Pharmacoeconomics (G. Liu et al.). The costs and effectiveness were both discounted annually at a rate of 5% (G. Liu, et al.). The incremental net health benefits (INHB) and incremental net monetary benefits (INMB) were also incorporated into our analyses using the following formulas: INHB (λ) = (μ*_E_*_1_ − μ*_E_*_0_) − (μ*_C_*_1_ − μ*_C_*_0_)/λ = Δ*E* − Δ*C*/λ; INMB (λ) = (μ*_E_*_1_ − μ*_E_*_0_) × λ − (μ*_C_*_1_ − μ*_C_*_0_) = Δ*E *× λ − Δ*C*, where μ*_Ci_* and μ*_Ei_* represented the costs and QALYs of cadonilimab for i = 1 or placebo for i = 0, respectively, while λ denoted the WTP threshold (Su et al., 2021). In order to assess the depreciation in value resulting from decision outcomes based on averages, we employed the expected value of perfect information (EVPI) to estimate the magnitude of losses resulting from suboptimal decision-making based on imperfect information (Bolam et al., 2019). The EVPI was calculated using the following formulas: Average (Max (NMB, cadonilimab, placebo)) − MAX (Average (NMB, cadonilimab, placebo)) (Rothery et al., 2020).

### 2.4. Sensitivity, scenario, and subgroup analyses

The robustness of the base-case results was assessed through one-way and probabilistic sensitivity analyses. In the one-way sensitivity analyses, we conducted a systematic adjustment of relevant parameters to their respective low and high values in order to identify key model input parameters that exerted substantial influence on the model outcome. The specific adjustments made are presented and visually depicted in Table 1. The ranges of the parameters utilized in the one-way sensitivity analyses were derived from reputable sources, or alternatively, a range equivalent to ±20% deviation from the base-case value was employed. Because healthcare negotiations in China solely lead to a reduction in drug prices, we set the price ceiling for cadonilimab ($875.02/120 mg) and bevacizumab ($158.37/100 mg) at their original costs. The probabilistic sensitivity analysis (PSA) was conducted by performing 1000 Monte Carlo simulations, with random sampling from the distributions of all parameters on each occasion. The assumed distributions were as follows: Gamma distribution for costs, Beta distribution for discount rate, utility values, and probabilities, and Log-normal distribution for HRs, body surface area, and weight. The cost-effectiveness acceptability curves and the EVPI were generated based on the results of 1000 iterations, aiming to better understand the uncertainty of CEA.

We subsequently performed a scenario analysis encompassing four conditions: employing parametric distributions for all survival curves, implementing price reductions of cadonilimab (at 50% or 30% of the original cost), varying time horizons within the model (10 or 20 years), and utilizing life-years as a measure of effectiveness.

Finally, to examine the impact of subgroup factors on economic outcomes, exploratory subgroup analyses were conducted for the predefined subgroups reported in the COMPASSION-16 trial by varying the HRs for OS and PFS.

## 3. Results

### 3.1. Base-case analysis

In comparison to the placebo group, treatment with cadonilimab resulted in an additional 1.18 QALYs and 1.52 overall life-years gained, at an incremental cost of $89,528.64. The cost attributed to first-line drug accounted for $88,709.40, constituting the majority of the incremental expenses. The ICER for cadonilimab vs placebo was estimated to be $75,944.56/QALY. The INHB was estimated to be −1.17 QALYs, while the INMB amounted to −$44,681.55. Furthermore, the EVPI was calculated as $71.40/person at a WTP threshold of $38,042.49/QALY (Table 2). The results obtained by utilizing parametric distributions for all survival curves were presented in Supplemental Table S2.

**Table 2.** Summary of cost and outcome results in the base-case analysis.

Variables	Cadonilimab	Placebo
Cost, $		
First-line drug	102,625.61	13,916.21
Overall	109,898.79	20,370.15
Life-years, year		
Progression-free	3.30	1.68
Overall	4.00	2.48
QALYs	2.87	1.69
ICER, $^a^		
Per life-year	58,924.65	
Per QALY	75,944.56	
INHB, QALY^a^	−1.17	
INMB, $^a^	−44,681.55	
EVPI/person, $	71.40	

Abbreviations: INHB, incremental net health benefit; INMB, incremental net monetary benefit; ICER, incremental cost-effectiveness ratio; QALY, quality-adjusted life-year; EVPI, expected value of perfect information.

^a^ Compared with Placebo strategy.

### 3.2. Sensitivity analysis

**Figure 2.**
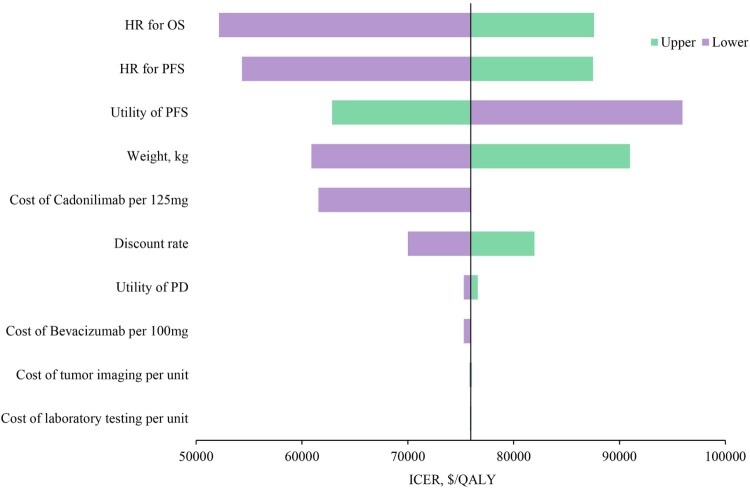
Tornado diagram of one-way sensitivity analyses of cadonilimab vs placebo.

**Figure 3.**
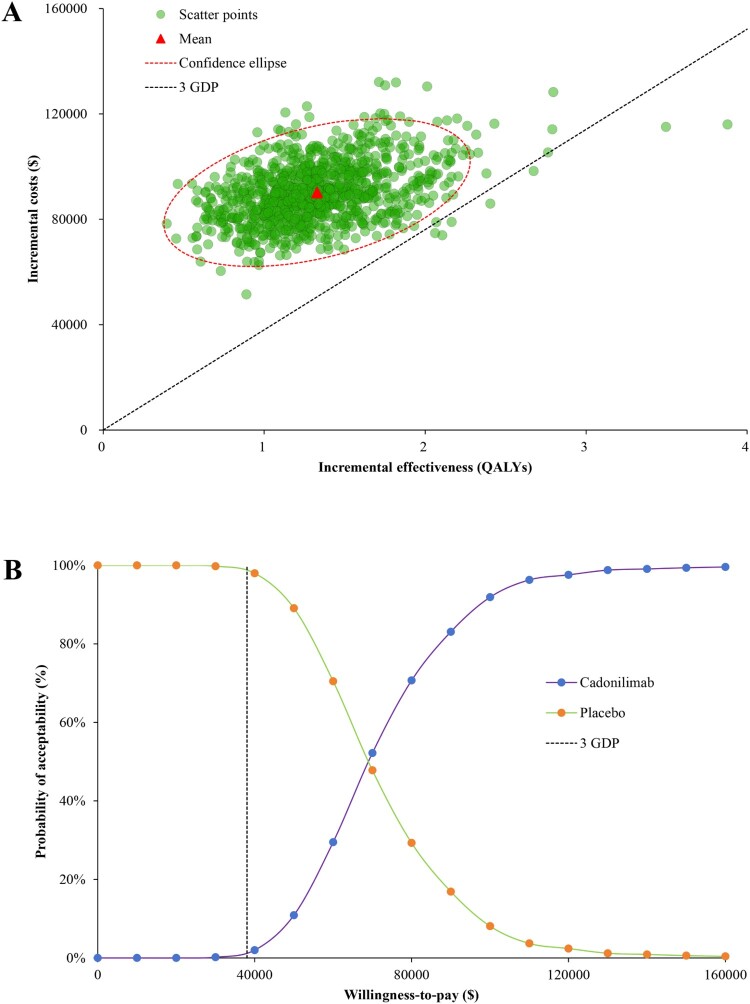
Probabilistic sensitivity analyses of cadonilimab vs placebo. (A) cost-effectiveness plane; (B) acceptability curves.

The one-way sensitivity analyses revealed that the HR for OS, HR for PFS, utility of PFS, cost of cadonilimab, and discount rate played crucial roles in the CEA outcomes (Figure 2). However, within the range of variation, no parameter can reverse the outcome. The results of the Monte Carlo simulation, consisting of 1000 iterations, indicated that cadonilimab was likely to exhibit superior efficacy but higher costs compared to the placebo. Notably, nearly 100% of iterations fell within the northeast quadrant on the cost-effectiveness plane (Figure 3(A)). As comparison to placebo, the 1000 iterations demonstrated that cadonilimab yielded an average increase of 1.33 QALYs (95% of values, 0.76-2.01), accompanied by an additional mean cost of $90,187 (95% of values, $72,898-$110,102), which resulted in a mean ICER of $72,950/QALY (95% of values, $46,483/QALY-$112,344/QALY), slightly lower than the baseline analysis outcome. The cost-effectiveness acceptability curve showed that the cadonilimab exhibited negligible economic advantages (0.7%) compared with placebo under pre-specified WTP threshold (Figure 3(B)).

### 3.3. Scenario and subgroup analyses

**Figure 4.**
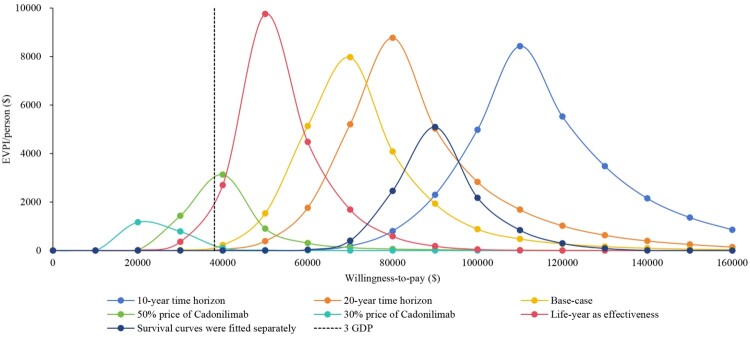
Value of information analysis results among different scenarios.

The scenario analysis demonstrated that the selection of survival analysis models and time horizons did not significantly impact the CEA results (Supplemental Table S3). The cost-effectiveness probability of cadonilimab would be 12.90% if the assessment considered effectiveness in terms of life-years rather than QALYs. The price scenario showed that the cadonilimab might be cost-effective with a probability of more than 52% when the price of cadonilimab was reduced by 50%. However, cadonilimab gained a positive INMB when the price of cadonilimab was reduced by 52.7% ($414/125 mg). If the price of cadonilimab decreases by 70% ($263/125 mg), there will be a probability exceeding 95% for it to achieve cost-effectiveness. When the WTP threshold fell within the range of $20,000 to $120,000, the majority of scenarios demonstrated their highest EVPI (Figure 4). By varying the HRs for OS or PFS, subgroup analyses revealed that the ICER of cadonilimab compared to placebo exceeded $40,000 per QALY, with a probability of being economically viable below 38% across all clinically relevant patient subgroups (Supplemental Table S4).

## 4. Discussion

As the economic burden of healthcare costs continues to increase, value-based oncology is receiving more attention, as are immunotherapy approaches (Monberg et al., 2024). The combination of immune checkpoint inhibitors (ICIs) and platinum-based chemotherapy has substantially changed the landscape for the treatment of cervical cancer. Following KEYNOTE-826 and BEATcc studies, the COMPASSION-16 phase 3 trial, adding cardonilizumab to platinum-based chemotherapy with or without bevacizumab significantly prolonged PFS and OS in these populations, suggesting promising clinical efficacy (Wu et al., 2024). However, this combination therapy involves substantial treatment costs, prompting us to evaluate its economic viability from the perspective of Chinese payers. To our knowledge, this is the first study to assess the economic outcomes of a PD-1 and CTLA-4 bispecific antibody in treating persistent, recurrent, or metastatic cervical cancer through the comprehensive analysis of the latest evidence using an economic modeling approach.

Our study addresses an unmet need for the economic evaluation of cadonilimab. Based on the results of the COMPASSION-16 trial, our analysis indicated that first-line cadonilimab plus chemotherapy with or without bevacizumab may not be a cost-effective treatment option for individuals with metastatic, persistent, or recurrent cervical cancer in China. According to the base-case analysis, cadonilimab plus chemotherapy cost $75,944.56 per additional QALY gained compared with placebo plus chemotherapy, nearly doubling the Chinese WTP threshold. The ability of cadonilimab to prevent disease-related death or progression was the major factor associated with economic outcomes. One-way sensitivity analysis demonstrated that the HR for OS and PFS were the most influential parameters, which is commonly seen in tumor immunotherapy (Su et al., 2021; Wan et al., 2019), as the ability of cadonilimab to prevent disease-related death or progression was the key factor driving economic outcomes. Cadonilimab was more likely to be cost-effective for patients with a favorable prognosis, such as those older than 65 years, without metastasis, no concomitant use of bevacizumab or prior chemoradiotherapy, and cisplatin use. This finding suggests that tailoring treatments based on individual patient factors can enhance economic outcomes, as studies on other malignant neoplasms have shown improved cost-effectiveness of immunotherapy in specific patient subgroups (Aguiar et al., 2017; Matter-Walstra et al., 2016; Su et al., 2021; Zargar et al., 2018). The body weight and cost of cadonilimab was also found to be important. Given that cadonilimab administration is weight-dependent, variations in body weight may result in disparate dosages per cycle, leading to substantial cost disparities. Consequently, further investigations are warranted to determine the optimal dosage of cadonilimab. Probabilistic sensitivity analysis estimated that cadonilimab plus chemotherapy had a mere 0.7% probability of being economically viable at the WTP threshold of $38,042.49/QALY, with viability exceeding 50% only at a WTP of about $75,000/QALY. Notably, when the cost of cadonilimab was reduced by 52.7% ($414/125 mg), it would achieve economic feasibility. However, across a wide range of each of these parameters, cadonilimab was still not cost-effective in China.

Several previously published studies evaluating the cost-effectiveness of chemotherapy in combination with other ICIs (pembrolizumab/atezolizumab) for the first-line treatment of recurrent or metastatic cervical cancer, demonstrated comparable results. A recent study (Lei et al., 2024) based on BEATcc trial revealed that chemotherapy combined with bevacizumab plus atezolizumab yielded an ICER of $295,972.43/QALY as compared to chemotherapy plus bevacizumab, which was not found to be cost-effective at the WTP threshold of $150,000/QALY in the United States, corresponding to findings from Zhu et al (Zhu et al., 2024). Reductions in the cost of atezolizumab by approximately 56% could make it cost-effective. Consistently, studies from Barrington et al (Barrington et al., 2022) and Shi et al (Shi et al., 2022) confirmed the results, who reported ICERs of $341,361 and $247,663 per QALY, respectively, far exceeding the WTP threshold in the United States, when comparing chemotherapy in combination with bevacizumab plus pembrolizumab with chemotherapy plus bevacizumab in recurrent or metastatic cervical cancer. Lin et al. (2024) also reported that pembrolizumab combined with chemotherapy and bevacizumab was not a cost-effective option in China. The difference in ICERs between these researches may be due to different modeling parameters, such as different costs, parametric survival functions, and utility values. Our conclusions are consistent with the above reports, although ICER varies greatly in different countries.

Although cadonilimab was approved for marketing in China by the National Medical Products Administration (NMPA) in June 2022, they are not currently covered by medical insurance. The high baseline costs of cadonilimab can lead to high out-of-pocket costs for patients, further increasing the economic burden and reducing accessibility. In recent years, the National Healthcare Security Administration has conducted several rounds of negotiations with pharmaceutical companies on the price of anticancer drugs, aiming to further reduce drug costs through national strategic procurement or bulk procurement. These initiatives may facilitate a reduction in the price of cadonilimab, potentially resulting in more favorable economic outcomes.

The analysis has several inherent limitations. First, the survival benefits beyond the follow-up period of the COMPASSION-16 trial were estimated by fitting parametric distributions or HRs, which may introduce uncertainty in the model outputs despite validation against observed data. Additionally, the estimation of PFS and OS is conducted independently. However, by disregarding the treatment effect, PSM may lead to an underestimation of extrapolated OS curves. Second, the utility values utilized in this model were derived from a cervical cancer CEA rather than directly estimated from the COMPASSION-16 trial, which may have implications for the generalizability and interpretation of our findings. The assumption of equal utility values in both groups may have potentially underestimated the efficacy of cadonilimab. However, the marginal variation in utility values would not have altered the cost-effectiveness balance as indicated by one-way sensitivity analyses. Third, the exclusion of grade 1/2 AEs in terms of cost and disutility may introduce potential biases in the estimation of cadonilimab benefits. Fortunately, the CEA outcomes remained robust to all parameters associated with AEs. Finally, the anti-tumor treatment assumed based on recommendation may exhibit slight variations from real-world practice due to limited information regarding disease progression.

## 5. Conclusion

In summary, from the perspective of Chinese healthcare system, the addition of cadonilimab to standard chemotherapy with or without bevacizumab is unlikely to be a cost-effective first-line option at the WTP thresholds of $38,042.49/QALY compared with chemotherapy for patients with persistent, recurrent, or metastatic cervical cancer. A substantial price reduction of cadonilimab may yield favorable economic outcomes considering its remarkable clinical efficacy. These findings will provide sound evidence for oncologists and policymakers to make optimal decisions and determine the value of different therapeutic alternatives in oncology.

## Supplementary Material

Supplementary Materials.pdf

## Data Availability

The dataset supporting the conclusions of this article is included within the article and its additional files.
