# Supplementary material for: Cost-effectiveness analysis of a first-line treatment with cadonilimab plus platinum-based chemotherapy with or without bevacizumab for persistent, recurrent, or metastatic cervical cancer in China: COMPASSION-16 trial
Source: J Pharm Policy Pract. 2025 Feb 17;18(1):2464781. doi: 10.1080/20523211.2025.2464781 (PMC11834776; doi:10.1080/20523211.2025.2464781)
Supplement: Supplementary Materials.pdf [file JPPP_A_2464781_SM9021.pdf]

Supplementary Materials

Supplementary Figure 1. The parametric K-M PFS Curves of Cadonilimab vs Placebo in the COMPASSION-16 trial.

Supplementary Figure 2. The Parametric K-M OS Curves of Cadonilimab vs Placebo in the COMPASSION-16 trial.

Supplementary Figure 3. The KM Curves of Cadonilimab in Proportional Hazards (PH) Model and Parametric Model

Supplementary Table 1. Evaluated Parameters and Values of AIC and BIC

Supplementary Table 2. Summary of Cost and Outcome Results Using Parametric Survival Functions

Supplementary Table 3. Summary of Cost and Outcome Results in Different Scenario

Supplementary Table 4. Results for Subgroup Analyses

Supplementary Figure 1. The parametric K-M PFS Curves of Cadonilimab vs Placebo in the COMPASSION-16 trial.

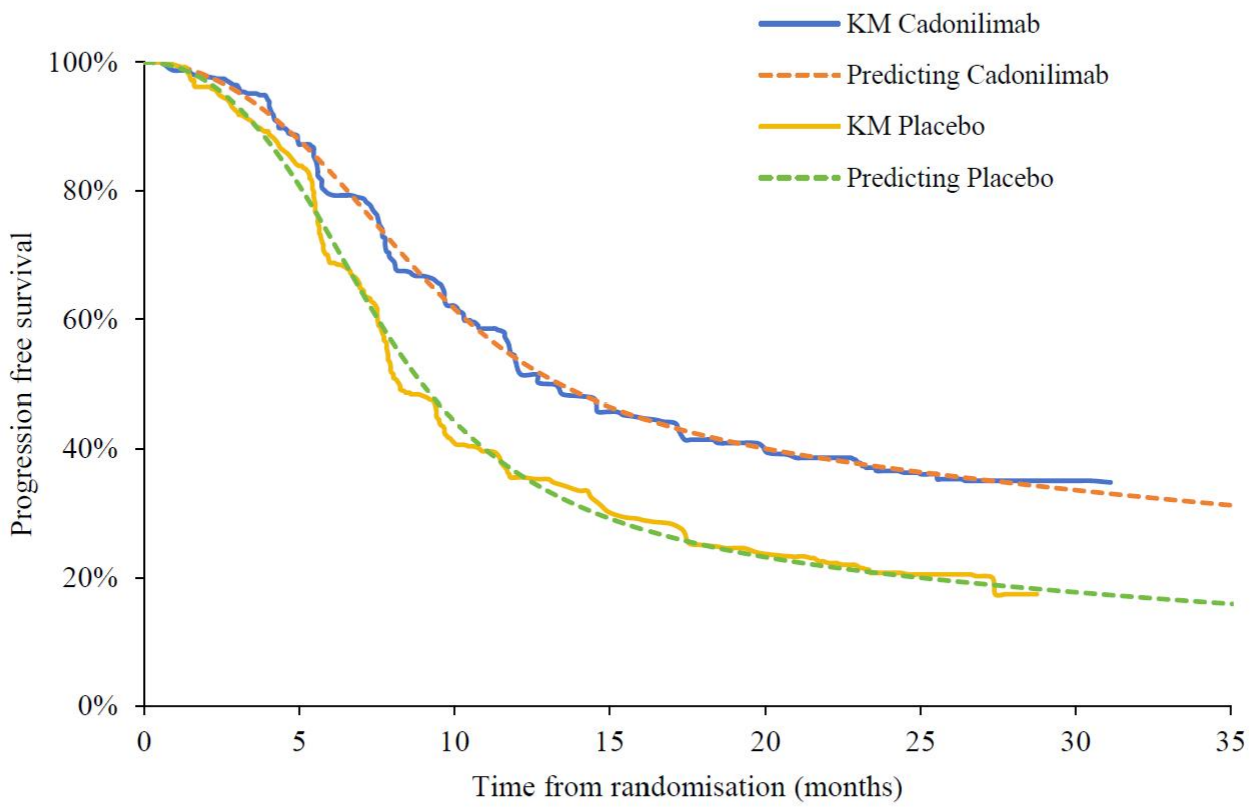

Supplementary Figure 2. The Parametric K-M OS Curves of Cadonilimab vs Placebo in the COMPASSION-16 trial.

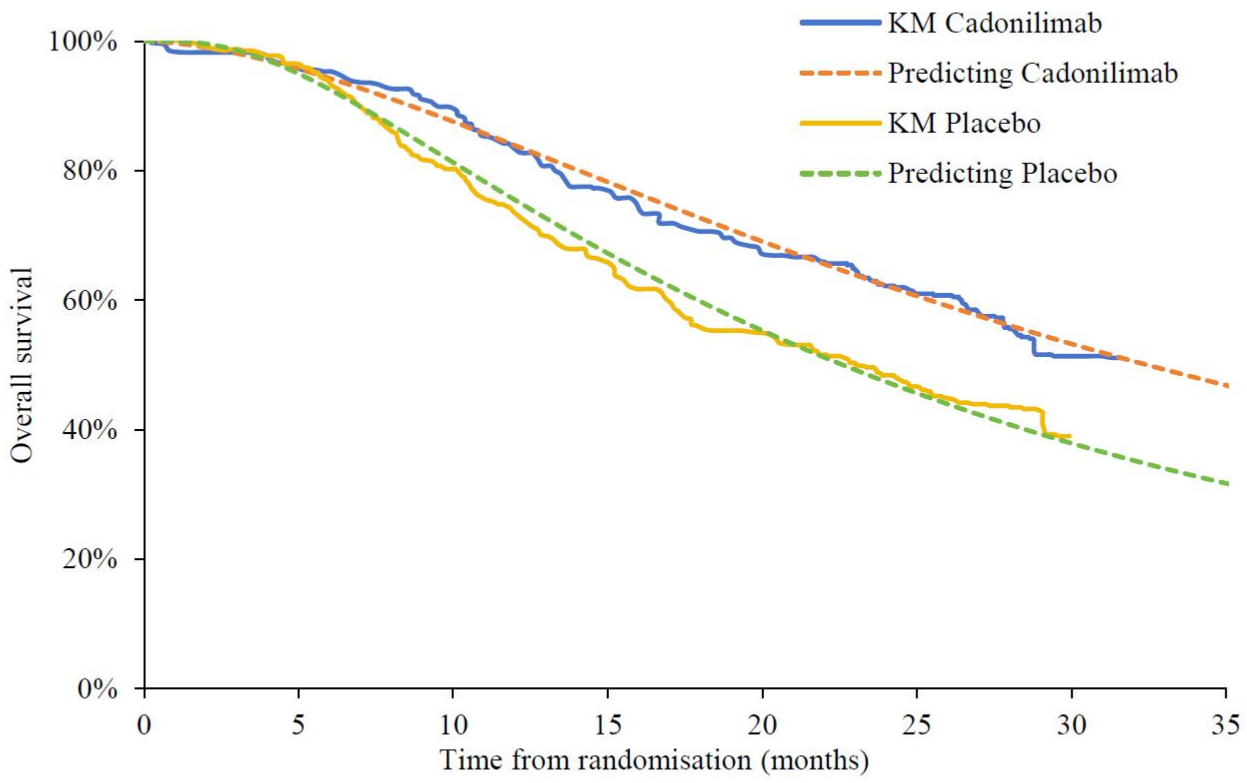

Supplementary Figure 3. The KM Curves of Cadonilimab in Proportional Hazards (PH) Model and Parametric Model

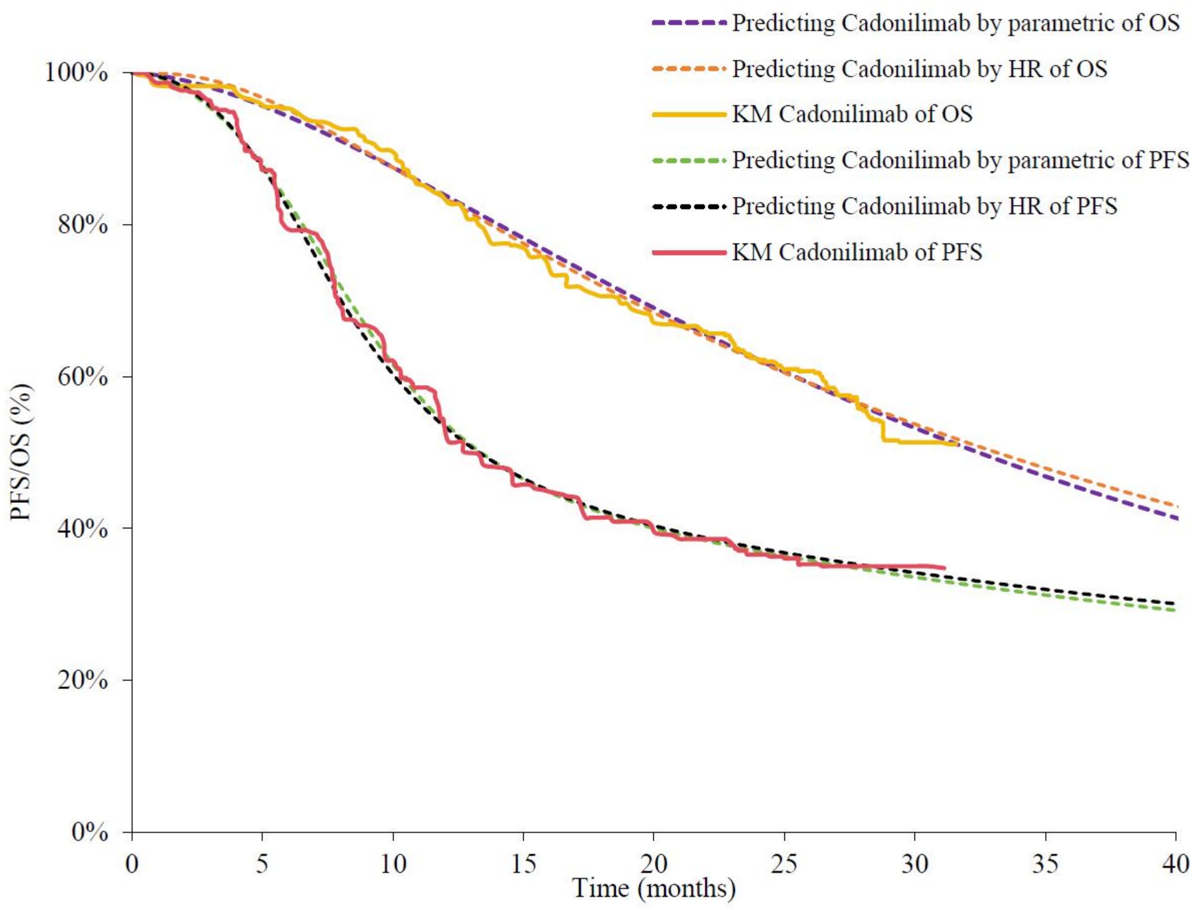

Supplementary Table 1. Evaluated Parameters and Values of AIC and BIC

Strategies	Distributions	Parameters	est	se	L95%	U95%	AIC	BIC
**Results of PFS**								
Cadonilimab	Exponential	rate	0.0416	0.0349	0.0496	0.0037	1038.725	1042.127
	Gamma	shape	1.4207	1.1432	1.7655	0.1575	1031.412	1038.218
		rate	0.0666	0.0486	0.0912	0.0107		
	Generalized gamma	mu	2.5925	2.2938	2.8912	0.1524	1016.045	1026.253
		sigma	1.1409	0.9839	1.3229	0.0862		
		Q	-0.3933	-0.9618	0.1752	0.2901		
	Gompertz	shape	-0.0042	-0.0291	0.0206	0.0127	1040.606	1047.412
		rate	0.0433	0.0323	0.0579	0.0064		
	Weibull	shape	1.2037	1.0377	1.3963	0.0911	1035.182	1041.987
		scale	22.7785	19.5977	26.4755	1.7480		
	Log-logistic	shape	1.5916	1.3751	1.8421	0.1187	1018.934	1025.739
		scale	15.2824	13.0266	17.9289	1.2453		
	Log-normal	meanlog	2.7565	2.5909	2.9220	0.0845	1015.833	1022.638
		sdlog	1.0756	0.9411	1.2293	0.0733		
	**Royston-Parmar（2 knot）**	gamma0	-5.2107	-6.6424	-3.7790	0.7305	**1003.785**	**1017.39**
		gamma1	1.9436	0.6997	3.1874	0.6346		
		gamma2	-0.6438	-1.2441	-0.0436	0.3063		
		gamma3	1.0688	0.3865	1.7510	0.3481		
Placebo	Exponential	rate	0.0671	0.0574	0.0784	0.0053	1171.823	1175.23
	Gamma	shape	1.6625	1.3629	2.0279	0.1685	1151.445	1158.259
		rate	0.1224	0.0947	0.1583	0.0160		
	Generalized gamma	mu	2.1469	1.9263	2.3675	0.1126	1127.631	1137.852
		sigma	0.9202	0.8203	1.0322	0.0540		
		Q	-0.4194	-0.8662	0.0273	0.2279		
	Gompertz	shape	0.0059	-0.0173	0.0292	0.0119	1173.574	1180.389
		rate	0.0639	0.0499	0.0819	0.0081		
	Weibull	shape	1.2839	1.1340	1.4537	0.0813	1160.021	1166.835
		scale	14.7702	13.0807	16.6778	0.9154		
	Log-logistic	shape	1.9317	1.6969	2.1991	0.1278	1128.898	1135.712
		scale	9.7618	8.6248	11.0487	0.6168		
	Log-normal	meanlog	2.3124	2.1855	2.4393	0.0648	1128.904	1135.718
		sdlog	0.8971	0.7994	1.0067	0.0528		
	**Royston-Parmar（2 knot）**	gamma0	-4.9688	-6.2155	-3.7221	0.6361	**1112.736**	**1126.364**
		gamma1	2.1035	1.0295	3.1776	0.5480		
		gamma2	-1.0286	-1.8599	-0.1973	0.4241		
		gamma3	1.3374	0.5148	2.1601	0.4197		
**Results of OS**								
Cadonilimab	Exponential	rate	0.0193	0.0156	0.0238	0.0021	852.966	856.369
	Gamma	shape	1.6307	1.2601	2.1103	0.2145	842.407	849.212
		rate	0.0414	0.0276	0.0621	0.0086		
	Generalized gamma	mu	3.6824	3.4830	3.8817	0.1017	844.323	854.531
		sigma	0.7294	0.4321	1.2313	0.1949		
		Q	0.8988	0.0672	1.7303	0.4243		

	Gompertz	shape	0.0423	0.0153	0.0693	0.0138	845.608	852.413
		rate	0.0111	0.0072	0.0173	0.0025		
	Weibull	shape	1.4553	1.1994	1.7657	0.1436	842.374	849.180
		scale	40.1260	33.5640	47.9709	3.6558		
	**Log-logistic**	shape	1.6517	1.3692	1.9925	0.1581	**842.186**	**848.992**
		scale	32.3136	26.9655	38.7224	2.9829		
	Log-normal	meanlog	3.5582	3.3364	3.7801	0.1132	848.509	855.315
		sdlog	1.1783	0.9987	1.3901	0.0994		
	Royston-Parmar（2 knot）	gamma0	-5.0788	-6.4149	-3.7428	0.6817	842.873	856.484
		gamma1	0.9764	0.1145	1.8383	0.4398		
		gamma2	-0.9027	-1.8344	0.0291	0.4754		
		gamma3	1.3822	-0.0572	2.8215	0.7344		
Placebo	Exponential	rate	0.0282	0.0233	0.0340	0.0027	988.831	992.238
	Gamma	shape	1.9278	1.5202	2.4448	0.2337	965.224	972.038
		rate	0.0704	0.0505	0.0983	0.0120		
	Generalized gamma	mu	2.8997	2.5656	3.2339	0.1705	956.152	966.373
		sigma	1.0070	0.8613	1.1775	0.0803		
		Q	-0.6407	-1.4438	0.1625	0.4098		
	Gompertz	shape	0.0360	0.0120	0.0600	0.0122	982.675	989.489
		rate	0.0184	0.0128	0.0264	0.0034		
	Weibull	shape	1.5175	1.2895	1.7858	0.1261	969.565	976.380
		scale	29.4533	25.7466	33.6937	2.0213		
	Log-logistic	shape	1.8603	1.5884	2.1788	0.1500	961.938	968.753
		scale	22.3007	19.3294	25.7288	1.6270		
	**Log-normal**	meanlog	3.1181	2.9675	3.2687	0.0769	**956.535**	**963.350**
		sdlog	0.9163	0.7927	1.0591	0.0677		
	Royston-Parmar（2 knot）	gamma0	-8.4505	-11.1874	-5.7136	1.3964	957.099	970.727
		gamma1	3.3431	1.8102	4.8760	0.7821		
		gamma2	0.4646	-0.7666	1.6958	0.6282		
		gamma3	0.0490	-1.3049	1.4029	0.6908		

Abbreviation: AIC, Akaike information criterion; BIC, Bayesian information criterion

Supplementary Table 2. Summary of Cost and Outcome Results Using Parametric Survival Functions

**Variables**	**Cadonilimab**	**Placebo**
Cost, $		
First-line drug	102921.20	13916.21
Overall	110008.37	20370.15
Life-years, year		
Progression-free	3.12	1.68
Overall	3.79	2.48
QALYs	2.71	1.69
ICER, $[a]		
Per life-year	68527.36	
Per QALY	87421.82	
INHB, QALY[a]	-1.33	
INMB, $[a]	-50631.24	
EVPI/person, $	0	

Abbreviations: INHB, incremental net health benefit; INMB, incremental net monetary benefit; ICER, incremental cost-effectiveness ratio; QALY, quality-adjusted life-years; λ, Willingness-to pay threshold.

[a] Compared with Placebo strategy.

Supplementary Table 3. Summary of Cost and Outcome Results in Different Scenario

Scenario	ICER	INHB	INMB	Probability of being cost-effective	EVPI/person
Base-case	75,944.56	-1.17	-44,681.55	0.70%	71.40
10-year time horizon	118,697.29	-1.59	-60,550.85	0.00%	0.00
20-year time horizon	84,695.72	-1.29	-49,250.10	0.30%	5.53
50% price of Cadonilimab	39,977.51	-0.06	-2,281.14	52.90%	3,646.33
30% price of Cadonilimab	25,590.69	0.39	14,679.03	95.80%	111.47
Life-year as effectiveness	58,924.65	-0.83	-31,727.84	12.90%	2,039.61
Parametric distributions for all survival curves	87,421.82	-1.33	-50,631.24	0.00%	0.00

Abbreviations: INHB, incremental net health benefit; INMB, incremental net monetary benefit; ICER, incremental cost-effectiveness ratio; EVPI, expected value of perfect information.

Supplementary Table 4. Results for Subgroup Analyses

Subgroup analysis	HR (95% CI) for OS	ICER, $/QALY	Probability of cost-effective	HR (95% CI) for PFS	ICER, $/QALY	Probability of cost-effective
**Overall**	0.65(0.49-0.87)	76651(53478-87985)	1.10%	0.62(0.49-0.79)	75945(54335-87500)	0.80%
Age						
<65 years	0.69(0.5-0.95)	79271(54839-90748)	0.50%	0.68(0.52-0.88)	81990(58843-90982)	1.00%
≥65 years	0.49(0.27-0.91)	53478(31129-89431)	11.30%	0.39(0.22-0.68)	41573(26708-81990)	37.10%
**ECOG performance status score**						
0	0.79(0.46-1.36)	84626(49606-99576)	0.30%	0.6(0.39-0.91)	72307(41573-91940)	1.30%
1	0.57(0.41-0.79)	65486(43806-84626)	3.20%	0.61(0.46-0.81)	74115(50144-88354)	1.20%
**Concomitant bevacizumab**						
Yes	0.84(0.56-1.26)	86804(63837-97981)	0.00%	0.78(0.57-1.06)	87056(67033-95304)	0.30%
No	0.5(0.33-0.75)	54839(35992-82662)	11.00%	0.44(0.31-0.63)	47526(33655-77792)	20.30%
**Previous concurrent chemoradiotherapy**						
Yes	0.84(0.56-1.26)	86804(63837-97981)	0.00%	0.78(0.57-1.06)	87056(67033-95304)	0.30%
No	0.5(0.33-0.75)	54839(35992-82662)	11.00%	0.44(0.31-0.63)	47526(33655-77792)	20.30%
**Pathological diagnosis**						
Squamous cell carcinoma	0.64(0.47-0.88)	75945(50862-88359)	1.40%	0.57(0.44-0.74)	67033(47526-85165)	1.50%
Non-squamous cell carcinoma	0.63(0.33-1.22)	75215(35992-97266)	1.70%	0.87(0.51-1.5)	90641(57306-95076)	0.30%
**Metastatic**						
Yes	0.84(0.56-1.26)	86804(63837-97981)	0.00%	0.78(0.57-1.06)	87056(67033-95304)	0.30%
No	0.5(0.33-0.75)	54839(35992-82662)	11.00%	0.44(0.31-0.63)	47526(33655-77792)	20.30%
**PD-L1 combined positive score**						
<1	0.77(0.44-1.34)	83671(47193-99277)	0.20%	0.65(0.42-1.03)	80255(45045-94811)	0.50%
≥1	0.69(0.49-0.97)	79271(53478-91363)	0.50%	0.62(0.47-0.83)	75945(51506-89162)	0.80%
≥10	0.68(0.42-1.08)	78645(44905-94317)	0.80%	0.54(0.37-0.79)	62022(39417-87500)	4.30%
**Cisplatin or carboplatin**						
Cisplatin	0.43(0.27-0.7)	46033(31129-79878)	23.20%	0.49(0.34-0.72)	54335(36410-84151)	8.40%
Carboplatin	0.82(0.57-1.18)	85966(65486-96499)	0.30%	0.72(0.53-0.97)	84151(60416-93562)	0.40%
